# Screening for Susceptibility-Related Biomarkers of Diclofenac-Induced Liver Injury in Rats Using Metabolomics

**DOI:** 10.3389/fphar.2021.693928

**Published:** 2021-09-23

**Authors:** Can Tu, Yuan Gao, Di Song, Ming Niu, Run-ran Ma, Ming-xi Zhou, Xian He, Xiao-he Xiao, Jia-bo Wang

**Affiliations:** ^1^ Beijing Research Institute of Chinese Medicine, Beijing University of Chinese Medicine, Beijing, China; ^2^ School of Traditional Chinese Medicine, Capital Medical University, Beijing, China; ^3^ China Military Institute of Chinese Medicine, Fifth Medical Center of Chinese PLA General Hospital, Beijing, China

**Keywords:** idiosyncratic drug-induced liver injury, diclofenac, susceptible individual, metabolomics, biomarker

## Abstract

Early identification of individuals susceptible to idiosyncratic drug-induced liver injury (IDILI) is a challenging unmet demand. Diclofenac, one of the most widely available over-the-counter drugs for pain management worldwide, may induce liver dysfunction, acute liver failure, and death. Herein, we report that diclofenac-related hepatobiliary adverse reactions occurred more frequently in cases with immune activation. Furthermore, experiments with rats demonstrated divergent hepatotoxicity responses in individuals exposed to diclofenac, and modest inflammation potentiated diclofenac-induced liver injury. Susceptible rats had unique plasma metabolomic characteristics, and as such, the metabolomic approach could be used to distinguish susceptible individuals. The 23 identified susceptibility-related metabolites were enriched by several metabolic pathways related to acute-phase reactions of immunocytes and inflammatory responses, including sphingolipid, tyrosine, phenylalanine, tryptophan, and lipid metabolism pathways. This finding implies a mechanistic role of metabolic and immune disturbances affects susceptibility to diclofenac-IDILI. Further nine metabolite biomarkers with potent diagnostic capabilities were identified using receiver operating characteristic curves. These findings elucidated the potential utility of metabolomic biomarkers to identify individuals susceptible to drug hepatotoxicity and the underlying mechanism of metabolic and immune disturbances occurring in IDILI.

## Introduction

Drug-induced hepatotoxicity is a major cause of severe internal damage, exhibiting divergent responses between individuals. Idiosyncratic drug-induced liver injury (IDILI) is the primary type of hepatotoxicity, causing liver dysfunction, acute liver failure, and death (Björnsson, 2016; Andrade et al., 2019). Unfortunately, except for drug cessation and liver transplantation, there are almost no effective treatments for IDILI. More importantly, the idiosyncratic nature of IDILI presents a vital challenge to its management due to the difficulty in predicting its incidence and the dosage of the causative pharmacotherapies (Roth and Lee, 2017; Uetrecht, 2019). In this regard, screening susceptible individuals using novel predictive biomarkers is of great value in the clinical prevention and management of this unique medicinal concern.

Emerging evidence shows that liver immunological and metabolic homeostasis remarkably affects susceptibility to IDILI (Chen et al., 2015; Hoofnagle and Bjornsson, 2019). Animal studies indicate that the destruction of immune homeostasis, triggered by inflammatory stress (Beggs et al., 2014; Tu et al., 2015) or the inhibition of immune tolerance (Chakraborty et al., 2015; Metushi et al., 2015), may enhance susceptibility to IDILI induced by hepatotoxic agents. It is well-known that dysimmunity may lead to an excessive inflammatory response in the liver, thereby causing hepatotoxicity. Strikingly, liver metabolic homeostasis also plays an essential role in IDILI susceptibility. A prospective study related to the hepatotoxicity of *Polygonum multiflorum* (PM) revealed that the overall serum profile comprising differential metabolic biomarkers could clearly distinguish susceptible patients before PM ingestion (Zhang et al., 2020). Moreover, previous studies have reported that several metabolites, such as succinic acid, potentially have a strong role in immune regulation (Mills et al., 2016). Therefore, the remodeling of metabolic homeostasis may drive significant immune dysfunction and thus cause the over sensitivity of the liver and consequently IDILI. The studies of IDILI susceptibility and the discovery of metabolic homeostasis biomarkers have been inconclusive. Metabolomics is a robust method that systematically evaluates metabolic homeostasis. Therefore, we believe using metabolomics may improve the prospects of identifying biomarkers related to IDILI susceptibility.

Diclofenac (Dicl), a non-steroidal anti-inflammatory drug (NSAID), is one of the most popular over-the-counter drugs worldwide for pain management in many diseases, including rheumatoid arthritis, ankylosing spondylitis, and osteoarthritis (Banks et al., 1995; Schmeltzer et al., 2016). However, IDILI is one of the main side effects of this drug. Previous studies report that Dicl may lead to acute liver failure and death, in addition to causing the elevation of hepatic transaminases. (Walker, 1997; Laine et al., 2009). Unfortunately, no research identifying individuals susceptible to Dicl-IDILI has been reported to date. Therefore, identifying predictive biomarkers for recognizing Dicl-IDILI–susceptible individuals, subsequently, preventing IDILI by avoiding drug use is an urgent issue to be addressed.

## Materials and Methods

### Chemicals and Reagents

Methanol and acetonitrile (HPLC grade) were bought from Thermo Fisher Scientific (Waltham, MA, United States). Other chemicals were all of analytical grade, and their purity was above 99.5%. Lipopolysaccharide (LPS, MFCD00164401) from *Escherichia coli* 055: B5 were obtained from Sigma–Aldrich (St. Louis, MO, United States). Alanine transaminase (ALT) and aspartate aminotransferase (AST) analysis kits were purchased from the Jiancheng Bioengineering Institute (Nanjing, China). Diclofenac (s80395-25 g) was purchased from Chengdu Chroma-Biotechnology Co., Ltd. (Chengdu, China).

### Adverse Drug Reaction–Related Database Source

Adverse drug reactions (ADRs) from January 1, 2012 to December 31, 2016 were obtained from the Chinese National Adverse Drug Reaction Monitoring System (ADRMS) database, China Food and Drug Administration. The hepatic ADR data were retrieved by searching for keywords in the ADRMS dataset (all in Chinese). The ADR reports recorded as liver injury related to ADRs, such as “drug-induced liver injury,” “drug-induced liver damage,” and “abnormal liver function caused by drugs,” as well as a medication history including Dicl (including different dosage forms), were included in this study. A retrospective survey method was used to analyze the demographic features (sex and age), clinical presentations (liver tests, underlying diseases, outcomes, and prognosis), medication information (drug variety, drug compatibility and combination, time of onset after starting the drug, and dosage), and ADR characteristics (reporting time, causality assessment of the ADR, and severity of the ADR).

### Experimental Design

Male Sprague–Dawley rats weighing 180 ± 20 g were provided by the Beijing Vital River Laboratory Animal Technology Co., Ltd. (certification number SCXK-(jing) 2016-0006). Room temperature and humidity were set to 20 ± 2°C and 60–70%, respectively. All rats were acclimated for 3 days before the experiments involving a 12-h day–night cycle and free access to a standard diet and water. For all the animal experiments in this study, Dicl was individually suspended in 0.5% CMC-Na and injected intraperitoneally (i.p.). Food and water were available ad libitum for all rats throughout the experiments. The study was approved by the Experimental Animal Ethics Subcommittee of Beijing University of Chinese Medicine (BUCM-4-2019091801-3067). The protocol was in accordance with the National Institute of Health Guide for the Care and Use of Laboratory Animals.

Based on previous experimental research (Deng et al., 2006), the rats were given Dicl (i.p.) at doses from 0 mg/kg to 100 mg/kg. In the initial animal experiment, rats were randomly assigned to six groups (n = 6 per group), each receiving a single i.p. injection of Dicl (0, 20, 30, 40, 50, or 100 mg/kg) and then killed after 12 h . Liver injury was evaluated by serum ALT activity and liver pathological assay.

In the primary animal experiments, rats were randomly allocated into four groups: the untreated control group (Con, n = 10); the non-toxic dose of the LPS model group (Mod, 2.8 mg/kg, n = 10); the low-dose Dicl group (Dicl, 20 mg/kg, n = 10); and the Mod model group in which rats treated with a low dose of Dicl (Mod/Dicl, n = 20). The experimental protocol was performed as previously reported (Tu et al., 2015). The rats were pretreated with LPS (2.8 mg/kg, i.v.) or medium. Two h later, the Dicl and Mod/Dicl groups were treated with Dicl (20 mg/kg, i.p.) or medium. Serum ALT and AST activity assays and liver pathological examinations were conducted 6 h after administration.

### Serum Biochemistry and Histopathology Analysis

Hepatic injury was estimated by evaluating serum ALT and AST activities using assay kits according to the manufacturer’s instructions. The liver samples were fixed with 10% neutral formalin for 48–72 h, embedded in paraffin after fixation, continuously sectioned at a thickness of 5 mm, stained with hematoxylin and eosin (H&E), and evaluated using a microscope. The histopathology analysis was performed with a Nikon E200 microscope (Nikon, Japan) at ×200 and ×400 magnification.

### Sample Preparation

Prior to the analysis, 300 µl serum samples (Con, Dicl, Mod, Mod/Dicl groups) were pipetted into individual 1.5-ml microcentrifuge tubes after being thawed at room temperature. All samples were extracted by adding 900 µl of acetonitrile, vortex-mixed, and centrifuged subsequently for 10 min at 14,000 rpm/min at 4°C. For metabolomic analysis, each supernatant was carefully separated into vials and filtered by a 0.22-µm microfiltration membrane.

### Chromatography and Mass Spectrometry Conditions

Chromatographic analysis was performed using an Agilent 6550 iFunnel Q-TOF LC/MS system (Agilent Technologies, United States). Samples were separated using an ACQUITY UPLC BEH-C18 column (2.1 × 100 mm, 1.7 μm) at 30°C. The mobile phase consisted of acetonitrile solvent A (0.1% formic acid in water) and solvent B (0.1% formic acid in acetonitrile), with an elution gradient set as follows: 0 ∼ 1 min, 5% A; 1 ∼ 9 min, 5 ∼ 40% A; 9 ∼ 19 min, 40 ∼ 90% A; and 19 ∼ 24 min, 90 ∼ 5% A. The flow rate was set at 0.30 ml/min, and the injection volume was 4 μl. To ensure stability and repeatability, a quality control (QC) sample was employed to optimize the UHPLC-Q-TOF/MS condition as it included the majority of the whole serum samples’ information. All the samples were held at 4°C during the experiment.

Mass spectrometry was performed using the electrospray ionization source (ESI) in both positive and negative modes. The detection parameters were as follows: electrospray capillary voltage: 3.5 kV (+) and 3.5 KV (−); the dry gas flow rate was 13 L/min; dry gas temperature was 225°C in the negative/positive ionization mode; nebulizer pressure was 20 pisg (negative) and 20 pisg (positive); sheath gas temperature was 275°C; the sheath gas flow rate was 12 L/min; and nozzle voltage was 2,000 V in both positive and negative modes. Data acquisition was performed in the full scan mode with a mass range between 80 and 1,500 m/z. After every five samples, a blank sample and a quality control (QC) sample were analyzed to ensure the stability and repeatability of the analysis.

### Identification of the Metabolites and Metabolic Pathway Analysis

To compare the metabolomic profiles, identified LC–MS data were processed with SIMCA-P™ software (version 14.0, published by UMetrics AB, Umea, Sweden) for principal component analysis (PCA) and orthogonal partial least-square discriminant analysis (OPLS-DA). Endogenous metabolites contributing to the classification were identified by the variable importance in the projection (VIP) values, which revealed the importance of each variable to the classification. Only VIP values *>* 1 were selected and used for further data analysis. Regarding the identification of biomarkers chosen due to a significant change (fold change (FC) *>* 1.5 or <0.5 and *p*-value < 0.05), the ion spectrum was matched with the structure message of the metabolites acquired from available biochemical databases, such as Mass Hunter PCDL Manager, HMDB, and KEGG. The pathway analysis of potential biomarkers was performed with MetaboAnalyst3.0 (http://www.metaboanalyst.ca/) to determine the relevant metabolic pathways. To further assess the sensitivity and specificity of the significantly differential metabolites, the receiver operating characteristic (ROC) curves for each metabolite were drawn, and the area under the curves (AUC) were calculated.

### Statistical Analysis

The experimental data were expressed as mean ± standard deviation and analyzed using SPSS 17.0 (SPSS Inc., Chicago, IL, United States). The intergroup variation was measured by a two–independent samples *t*-test. The normality and homogeneity of variance were tested. If all the data were satisfied, then groups of independent sample *t*-tests were applied. If not, a non-parametric test was required (Mann–Whitney U test, n < 50). The difference was considered statistically significant when *p* < 0.05.

## Results

### Dicl-Related Hepatobiliary ADR Frequently Occurred in Cases With Immune Abnormalities

A total of 160 hepatobiliary ADR cases associated with Dicl (including different dosage forms) were identified in the ADRMS database (2012–2016) and included in this study. The ratio of female to male patients was 1:1.67, and the median age of onset was 52 years (range, 4–86 years). In total, 100 male (62.5%, median age: 49 years) and 60 female (47.5%, median age: 54 years) patients were included. The age distribution revealed that 3.8, 21.9, 40.0, and 34.3% of ADR cases appeared among those less than 19 years, 20–39 years, 40–59 years, and above 60 years, respectively (Supplementary Table S1).

**FIGURE 1:**
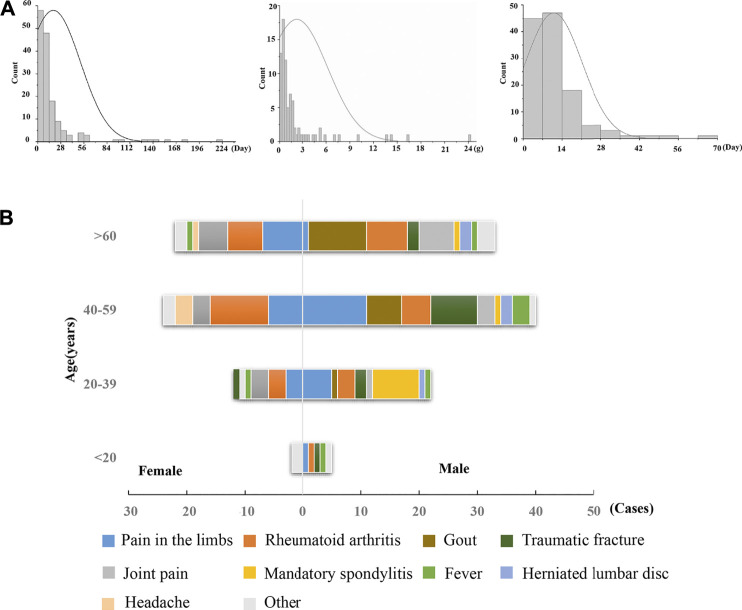
Characteristics of Dicl-related hepatobiliary ADR. **(A)** Time of hepatobiliary ADR, accumulated dose, and improvement time of Dicl-related hepatobiliary ADR. **(B)** Sex, age, and underlying complications of Dicl-related hepatobiliary ADR.

Notably, there was no obvious dose–toxicity relationship between the cumulative dosage and the duration of administration (Figure 1A), which is a typical phenomenon of IDILI. There were 116 cases (72.5%) with general underlying complications, including limb pain, gout, rheumatoid arthritis, joint pain, and traumatic fracture. The predominant symptoms included limb pain and rheumatoid arthritis for both male and female patients. However, male patients also commonly experienced gout, while females reported joint pain. In addition, Dicl-hepatobiliary ADR often occurred in cases with immune abnormalities (Figure 1B), such as with gout, rheumatoid arthritis, and joint pain, which suggested that abnormal immune activation in the hosts might be a factor increasing susceptibility to Dicl-IDILI.

### Modest Immune Activation Potentiated Dicl-IDILI in Rats

**FIGURE 2:**
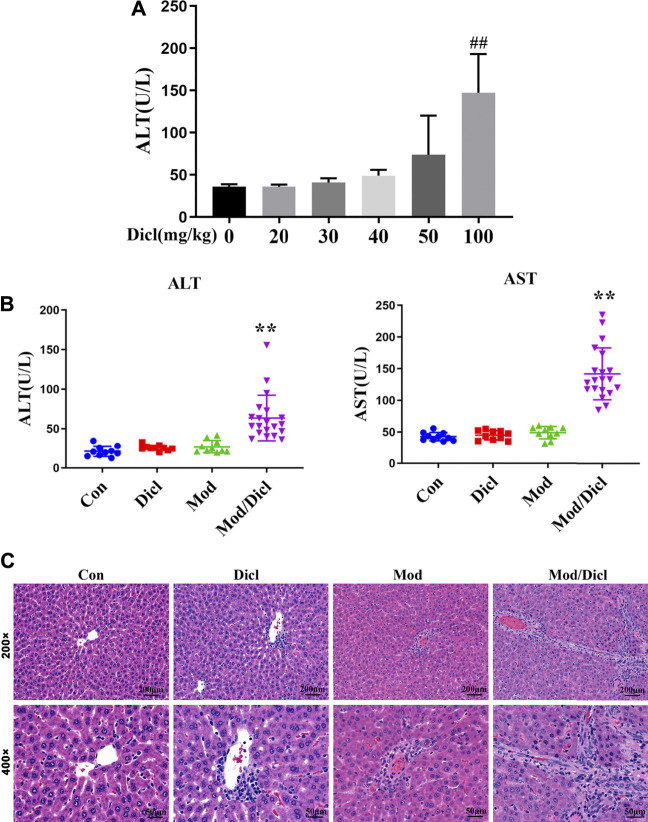
Phenotypes of Dicl-IDILI in normal rats or model rats with modest inflammation. **(A)** Dose-responsive liver injury phenotype of Dicl exposure in normal rats. Dicl doses ranged from 0 to 100 mg/kg (i.p.) (n = 6, ^##^ *p* < 0.01 *vs*. control). **(B)** The liver injury phenotype of Dicl (20 mg/kg, i.p.) exposure in rats with modest inflammation. Con (n = 10), the control group; Mod (n = 10), the non-toxic dose of the LPS-induced modest inflammation model group; Dicl (n = 10), normal rats treated with a low dose of Dicl (20 mg/kg, i.p); Mod/Dicl (n = 20), the modest inflammation model of rats treated with a low dose Dicl (20 mg/kg, i.p). The results are expressed as mean ± SD, and significant differences are indicated (^∗∗^ *p* < 0.01, *vs.* Mod). **(C)** Histological alterations in rat livers among the different groups (H&E stained, ×200 and ×400 magnification).

The administration of 100 mg/kg Dicl caused a significant increase in ALT activity within 12 h (Figure 2A), suggesting that large doses of Dicl can cause liver injury. According to the aforementioned ADRMS database, Dicl-IDILI more frequently occurred in cases involving immune activation. We thus evaluated whether modest immune activation increased susceptibility to Dicl-IDILI in a non-toxic immune activator (LPS)–induced modest immune activation rat model (Beggs et al., 2014; Tu et al., 2015). Treatment with a low dose of Dicl (20 mg/kg) or a non-toxic dose of LPS alone did not cause a significant increase in serum ALT and AST activities in rats (*p* > 0.05), compared with the control group. By contrast, serum ALT and AST activities showed significant increases in the Mod/Dicl group, compared with the susceptibility model group (*p* < 0.05). Taken together, modest immune activation potentiated Dicl-induced liver injury in rats, implying that it increases susceptibility to Dicl-IDILI (Figure 2B).

In addition, histological analysis showed trends similar to serum liver biochemistry. Specifically, rat livers from both the control and Dicl-treated groups did not show histological changes (Figure 2C), while livers from the LPS-treated groups revealed mild infiltration of inflammatory cells, occasional hepatocellular apoptosis, and modest parenchymal edema. By contrast, livers from the Mod/Dicl group enhanced hepatocellular apoptosis, parenchymal edema, cell invasion in the portal area around the blood vessels, and a small amount of Kupffer cell infiltration, which indicated significant activation of inflammatory signaling pathways.

### Metabolomic Profile Analysis

**FIGURE 3:**
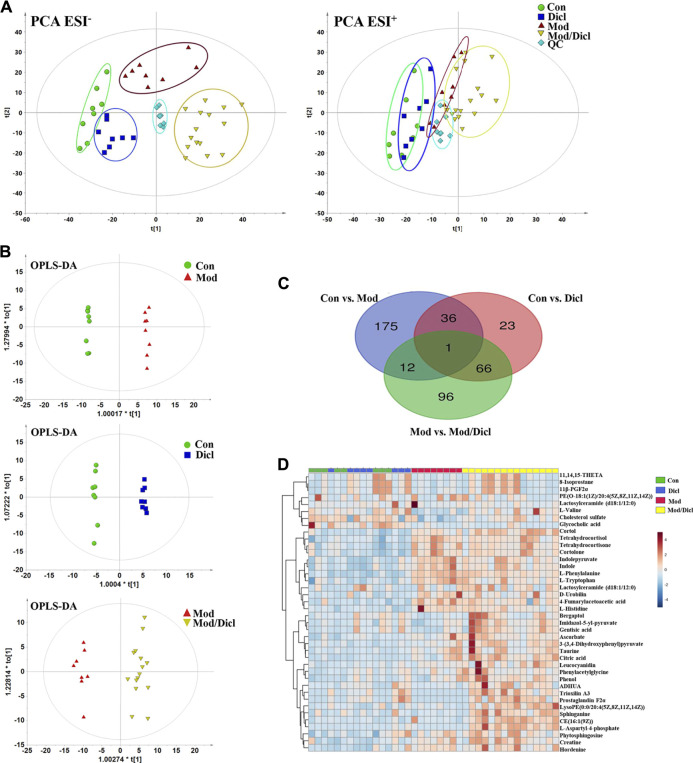
Metabolomic profile analysis of Dicl-IDILI in rats. **(A)** The PCA score plots for different experimental groups of either negative (left) or positive (right) ESI modes, respectively. The rats were randomized into four groups as follows: the Con group (n = 8), Dicl group (n = 8), Mod group (n = 8), and Mod/Dicl group (n = 15). **(B)** OPLS-DA score plots of the paired groups (Con *vs*. Mod, Con *vs.* Dicl, and Mod *vs*. Mod/Dicl). **(C)** The number of shared and unique metabolites visualized in the Venn diagram for Con *vs.* Mod, Con *vs.* Dicl, and Mod *vs.* Mod/Dicl. **(D)** The clustered heat map of the 40 metabolites with significantly different expressions among the control, Mod, Dicl, and Mod/Dicl groups. The colors in the heat map indicate increased (red) or decreased (blue) relative metabolite contents.

Score plots from the PCA derived from the ESI^−^ and ESI^+^ modes are shown in Figure 3A. An unsupervised PCA statistical method was employed to assess the metabolic differences between groups. The QC samples were distributed centrally and clustered near the middle of the scoring matrix projection graph, indicating the robustness of the metabolomics methodology throughout the analysis. In PCA plots of both ESI^−^ and ESI^+^ modes, obvious distances were observed among the control, Mod, Dicl, and Mod/Dicl groups, which suggests immune activation– or drug exposure–dependent metabolic profiles. The Mod/Dicl group was farthest from the other groups, which was consistent with the liver injury phenotype in serum biochemistry and histopathology.

The supervised statistical method of OPLS-DA plots was performed to further screen potential candidate biomarkers associated with the susceptibility to Dicl-IDILI. As shown in Figure 3B, there were remarkable separations among Con *vs*. Mod, Con *vs*. Dicl, and Mod *vs*. Mod/Dicl. On this basis, variables that expressed differently between the two groups were screened out using either multivariate or univariate statistical significance criteria (VIP>1, FC > 1.5 or <0.5, *p* < 0.05). Venn diagram analysis showed that there were 175 (Con vs. Mod), 96 (Mod vs. Mod/Dicl), and 23 (Con vs. Dicl) differential ions within these independent comparisons, which were then identified by the accurate mass-to-charge ratio in the Mass Hunter PCDL Manager online database (true mass tolerance mass error <10 ppm) and MS/MS fragment patterns (Figure 3C). In total, 40 susceptibility-related and liver injury–related metabolites with significantly different expressions were identified from the abovementioned 271 ions **(** Supplementary Table S2, S3). From the heat map cluster analysis, changes in the metabolites screened by OPLS-DA revealed different expression patterns between the groups (Figure 3D).

### Screening for Susceptibility-Related Biomarkers

**FIGURE 4:**
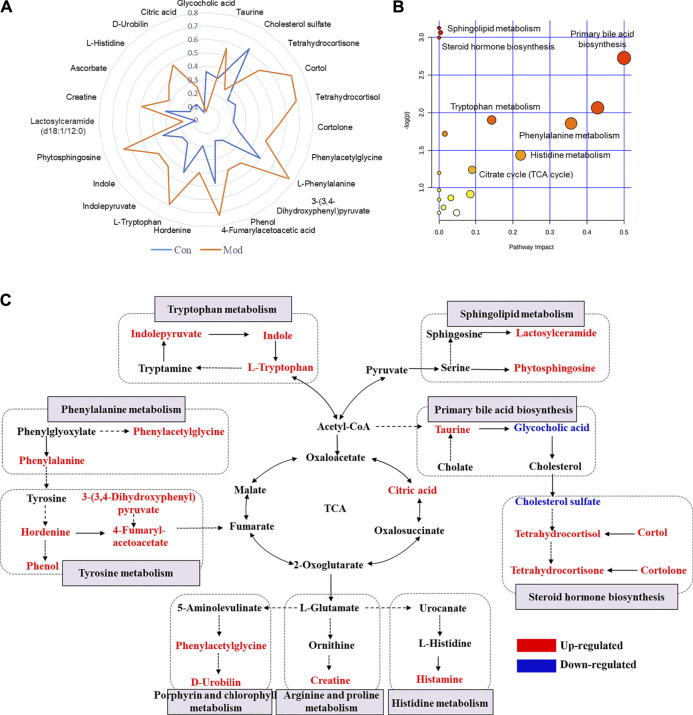
Profile of susceptibility-related metabolomic biomarkers of Dicl-IDILI. **(A)** The relative abundance radar plot of 23 metabolites. The blue line represents the control group (Con), and the orange line represents the susceptibility model group (Mod). **(B)** The bubble diagram of the disturbed metabolic pathways. **(C)** The network map of metabolic pathways and metabolites. Metabolites in red or blue indicate up- or downregulated expressions, respectively, in the Mod group compared to the control group.

Considering that the modest inflammation induced by non-toxic LPS potentiates susceptibility to Dicl-IDILI, we screened the susceptibility-related biomarkers by comparing the differences between the control group and the susceptibility model (Mod) group. Twenty-three metabolites were found (Supplementary Table S2). To further compare the differences between the control and Mod groups, a radar plot of those metabolites with significantly different expressions was constructed based on their relative abundances (Figure 4A). Compared with the control group, the differential metabolites of the LPS-induced immune activation group (Mod group) showed significant upregulation, except for glycocholic acid and cholesterol sulfate. A schematic diagram of the disturbed metabolic pathways is presented in Figure 4B to summarize the metabolic disorder of the susceptibility model. A network map was constructed based on the identified metabolites with significantly different expressions between the two groups and then enriched into specific metabolic pathways (Figure 4C). Compared with the control group, there were significant differences in sphingolipid, tyrosine, phenylalanine, tryptophan, primary bile acid biosynthesis, and steroid hormone biosynthesis metabolism pathways in the susceptibility model.

**FIGURE 5:**
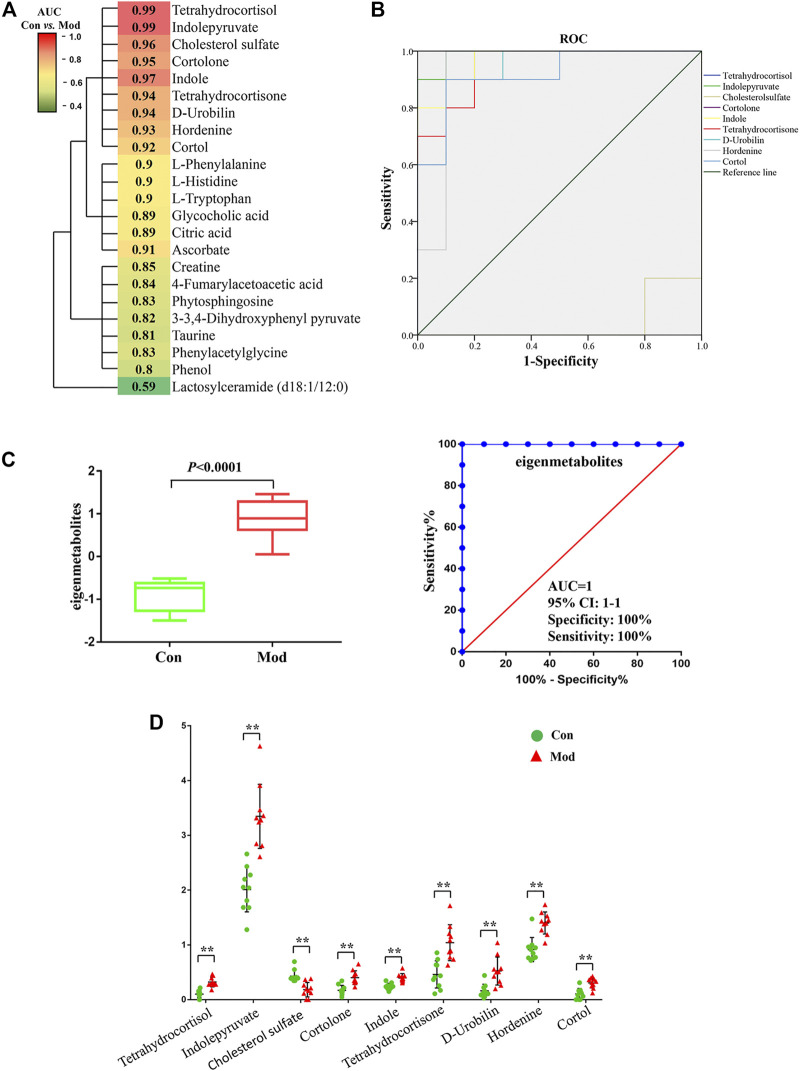
Susceptibility-related metabolite biomarkers of Dicl-IDILI. **(A)** Cluster analysis of the area under the curve (AUC) and *p*-values of the receiver operating characteristic (ROC) curve of each of the 23 metabolites in discriminating the susceptibility model (Mod) group from the control (Con) group. The color indicates the AUC value. **(B)** ROC curves of nine metabolites in discriminating the Mod group from the Con group. **(C)** Identification of the susceptible-related metabolic fingerprint (eigenmetabolite). **(D)** Differential expressions of nine metabolites in the Mod group and the Con group (^∗^ *p* < 0.05,^∗∗^ *p* < 0.01 *vs.* Con).

Most of the 23 candidate biomarkers revealed good diagnostic effectiveness with a high AUC observed in the ROC curves (all >0.8), except for one metabolite, lactosylceramide (d18:1/12:0; AUC <0.6; Figure 5A). According to the clustering analysis using the AUC and *p*-values of each metabolite, nine metabolites clustered into one group had the highest diagnostic effectiveness (AUC >0.9 and *p* < 0.001). The ROC curves of the nine metabolites (tetrahydrocortisol, indolepyruvate, indole, etc.) are depicted in Figure 5B, and the group differences of the nine metabolites are shown in Figure 5D. Next, we computed the eigenmetabolites of these 9 metabolites by PCA and found a significant increase in eigenmetabolites from the control and model groups. We found that the susceptible-associated metabolic fingerprint (eigenmetabolite) had better capability of identifying susceptible individuals than those significant metabolites (AUC values 1 *vs.* 0.9, Figure 5C). Collectively, these findings indicate that the 9-metabolite cluster can serve as a fingerprint of serum metabolites, which characteristically differentiated susceptible and normal groups.

### Screening for Hepatotoxicity-Related Biomarkers

Similarly, we screened potential hepatotoxicity-related biomarkers of Dicl-IDILI. There were 17 metabolites with significantly different expressions between the Mod and Mod/Dicl groups (Supplementary Table S3; Supplementary Figure.S1A). The pathway enrichment results showed that the remodeling of those metabolic pathways, such as the arachidonic acid, sphingolipid, and tyrosine metabolic pathways, had a close relationship with the Dicl-IDILI phenotype in biological networks (Supplementary Figures S1B,C).

The differentiating capability of the 17 metabolites was assessed by ROC curves followed by cluster analysis. There were five metabolites clustered into one group, which had good diagnostic effectiveness (AUC ≥0.9 and *p* < 0.001; Supplementary Figure S2A). In Supplementary Figure S2B, these five metabolites were significantly increased in the Mod/Dicl group, compared to either the model or control groups, which might be of potential utility for identifying Dicl-IDILI individuals and in clinical safety management.

## Discussion

Due to the great variation in toxic responses and the unpredictable nature of IDILI, early identification of susceptible individuals is critically important in clinical practice. In this study, we found that low doses of Dicl (20 mg/kg) induce liver injury in non-toxic immune activator–induced modest immune activation model rats. In contrast, Dicl only causes liver injury at high doses (100 mg/kg) in normal rats, which is a five-fold exposure compared to the susceptibility model rats (Figure 2). These phenotypes imply that modest immune activation is an important factor influencing susceptibility to Dicl-IDILI. By a whole-spectrum untargeted metabolomics approach, 23 plasma metabolites were identified to have a close relationship with susceptibility, and 17 metabolites were found to be associated with liver injury. The 23 identified susceptibility-related metabolites were enriched by several metabolic pathways related to acute-phase reactions of immunocyte and inflammatory responses. These observations imply that a mechanistic role of metabolic and immune disturbances affects susceptibility to Dicl-IDILI (Figures 4, 5). Taken together, our findings elucidate the potential utility of metabolomics for identifying hepatotoxic drug susceptibility to IDILI and the underlying mechanism of metabolic and immune disturbances.

Previous studies have suggested that metabolic factors, oxidative stress, gene polymorphism, and mitochondrial injury may play a pathological role in Dicl-IDILI (Syed et al., 2016; Oda et al., 2017). In a study on rats with Dicl-IDILI, serum miR-122 was found to be a more specific biomarker of hepatotoxicity than liver transaminases (Sharapova et al., 2016). Another study reported that chemokine receptors CCR2 and CCR5 promoted the pathophysiological process of Dicl-IDILI (He et al., 2019). Furthermore, levels of IL-10 and IL-4 gene polymorphism can increase susceptibility to Dicl-IDILI (Aithal et al., 2004). Overall, evidence implies that immune factors are important contributors to Dicl-IDILI. In the current study, Dicl-IDILI had no obvious dependence on the dose or course of treatment (Figure 1), which is a typical characteristic of IDILI. Moreover, many cases had comorbidities, including rheumatoid arthritis, joint pain, and gout, which involve abnormal immune activation or elevations in pro-inflammatory cytokines (Chen et al., 2019). These results suggest that patients might become susceptible to Dicl-IDILI during the destruction of immune homeostasis. Furthermore, we also confirmed that a single therapeutic dose of Dicl (20 mg/kg) resulted in abnormal liver biochemical indicators and histological liver damage in modest inflammation model rats but caused no liver injury in normal rats. Therefore, it could be deduced that modest immune activation potentiates susceptibility to Dicl-IDILI.

Since the metabolic and immune systems are interdependent and highly integrated (Prochnicki and Latz, 2017), we considered the utility of the metabolomics approach to depict the characteristic profile of individuals susceptible to Dicl-IDILI. Our results confirm that the disturbance to immune homeostasis caused by modest immune activation can be successfully characterized by a panel of metabolites. Recent studies have shown that metabolic reprogramming is an important basis for the development, proliferation, and functional phenotype of immune cells (Kishton et al., 2017). Therefore, our study illustrates the utility of metabolomics to understand and identify disturbances in immune homeostasis from the perspective of metabolic reprogramming. This unique metabolic homeostasis reprogramming may play a vital role in affecting susceptibility to IDILI. Metabolic homeostasis remodeling may drive immune dysfunction, leading to the liver becoming more sensitive to drug toxicity, thereby increasing the risk of hepatotoxicity.

Further pathway enrichment analysis of Dicl-IDILI susceptibility showed that phenylalanine, tyrosine, tryptophan, primary bile acid biosynthesis, and sphingolipid metabolism were the main metabolomic characteristics distinct in the susceptibility model group. Amino acid metabolism plays a vital role in immune and inflammatory reactions, synthesizing proteins involved in immune cell proliferation and immune reactions (Prochnicki and Latz, 2017). These aromatic amino acids (such as phenylalanine, tyrosine, and tryptophan) are predominant in acute-phase proteins (Tu et al., 2019), which may be related to a large amount of acute protein synthesis in the inflammatory stress state. It has been reported that when the host is stimulated or damaged by inflammation, monocytes and macrophages abundantly secrete pro-inflammatory cytokines, which mediate the synthesis and secretion of acute proteins, thus causing acute-phase reactions. Moreover, evidence from both clinical and experimental studies shows the overexpression of kynurenines, which are involved in the tryptophan’s metabolic processes, may contribute to hepatopathy (Wirthgen et al., 2017). Compared to the control group, the contents of indolepyruvate, indole, and tryptophan were significantly increased in the susceptibility model group. These results indicate an abnormal metabolic process of tryptophan, but it remains unclear which part of the abnormality has occurred. In addition, histamine is a well-known inflammation mediator that is released from mast cells and basophils that enable the regulation of various functions by the production of cytokines and chemokines (Hirasawa, 2019). Compared to the control group, histamine content was significantly increased in the susceptibility model group. These results suggest that disordered histidine metabolism also caused the destruction of metabolic and immune homeostasis. In the current study, we also identified elevated phytosphingosine and lactosylceramide serum concentrations as potential new biomarkers related to Dicl-IDILI susceptibility. Some sphingolipid ceramides, including sphingosine 1-phosphate and ceramide 1-phosphate, have been widely implicated in immune signal responses and transductions, such as TNF-α secretion by a feedback mechanism (Gomez-Muñoz et al., 2016). Overall, susceptibility to Dicl-IDILI may be closely related to disturbed metabolic and immune homeostasis.

Furthermore, based on the susceptibility factors identified with the modest inflammation activation model, low doses of Dicl caused significant liver injury, accompanied by the inflammatory response. The immune abnormalities in Dicl-IDILI mainly showed that upregulation of the arachidonic acid, sphingolipid, tyrosine, and bile secretion metabolic pathways had a close relationship with Dicl-IDILI (Serhan and Savill, 2005; Korotkova and Lundberg, 2014; Sonnweber et al., 2018). In this study, the significant changes in trioxilin A3, 11β-PGF2α, PGF2α, and 11, 14, and 15 THETA from arachidonic acid might be associated with the abnormal immune response because an increased level of arachidonic acid metabolites (which become the precursor of pro-inflammatory bioactive mediators) results in inflammatory damage to the liver. Bile acids also act as damage-related molecular models (DAMPs), which cooperatively participate in the activation of signaling pathways during the initiation and activation stages of NLRP3 inflammasomes, secreting pro-inflammatory cytokine IL-1β and inducing the inflammatory response (Guo et al., 2016).

In summary, this study demonstrated the idiosyncratic characteristics of Dicl-induced liver injury from analysis of the ADRMS database and animal models of modest inflammation. Based on immune inflammation and the metabolic disorder pathways associated with susceptibility factors, we found 9 metabolites related to inflammation and immune regulation (such as tetrahydrocortisol, indolepyruvate, and indole) as potential biomarkers for identifying susceptible model animals (ROCAUC >0.9). Although the biomarkers highly correlated with Dicl-IDILI were screened through metabolomics, which can provide some reference for the mechanism and clinical practice, it needs to be validated by future prospective clinical trials before it can predict the susceptible individuals of Dicl-IDILI. Meanwhile, it is necessary to distinguish the specific biomarkers of IDILI between Dicl and other IDILI drugs, so as to find unique biomarkers of Dicl-IDILI. Finally, the ADRMS database and pathway enrichment analysis indicated that mild inflammation and metabolic disorder may be involved in Dicl-IDILI, and in-depth mechanism verification is required. This study also provides a new perspective for understanding the mechanism of susceptibility to IDILI and guides the clinical safety management of drugs causing IDILI.

## Data Availability

The original contributions presented in the study are included in the article/Supplementary Material; further inquiries can be directed to the corresponding author.

## Supplementary Material

Supplementary Figure S1 Profile of hepatotoxicity-related metabolomic biomarkers of Dicl-IDILI. (A) The radar plot of 17 hepatotoxicity-related metabolites showing evidence of the different metabolic profiles between the Mod and Mod/Dicl groups. (B) Schematic diagram of the disturbed metabolic pathways. (C) Network map of metabolic pathways and metabolites.

Supplementary Figure S2 Hepatotoxicity-related metabolite biomarkers of Dicl-IDILI. (A) Cluster analysis on the AUC and p-values from the ROCs of each of 17 metabolites in discriminating the Dicl-IDILI (Mod/Dicl) group from the susceptibility model (Mod) group. The color indicates the AUC value. ADHUA: 2-Amino-3,7-dideoxy-D-threo-hept-6-ulosonic acid; LysoPE: LysoPE [0:0/20:4(5Z,8Z,11Z,14Z)]. (B) Differential expressions of 4 metabolites in the Mod/Dicl and Mod groups (*p < 0.05, **p < 0.01 vs. Mod).

## Abbreviations

IDILI, idiosyncratic drug-induced liver injury; Dicl, diclofenac; ROC, receiver operating characteristic; PM, *Polygonum multiflorum*; NSAIDs, non-steroidal anti-inflammatory drug; ALT, alanine transaminase; AST, aspartate aminotransferase; ADR, adverse drug reaction; H&E, hematoxylin and eosin; PCA, principal component analysis.
