# Supplementary material for: Screening for Susceptibility-Related Biomarkers of Diclofenac-Induced Liver Injury in Rats Using Metabolomics
Source: Front Pharmacol. 2021 Sep 23;12:693928. doi: 10.3389/fphar.2021.693928 (PMC8494976; doi:10.3389/fphar.2021.693928)
Supplement: Supplementary file 1 [file Table1.DOCX]

**Table S1 Clinical Characteristics of the Dicl-related hepatobiliary ADR Cases**

| Characteristic | Male (n=100) | Female (n=60) | Total (n=160) |
| --- | --- | --- | --- |
| Median age (yr), n(%) | 49(6-83) | 54(4-86) | 52(4-86) |
| <18 | 4(4.0) | 2(3.3) | 6(3.8) |
| 19~39 | 23(23.0) | 12(20.0) | 35(21.9) |
| 40~59 | 40(40.0) | 24(40.0) | 64(40.0) |
| >60 | 33(33.0) | 22(36.7) | 55(34.3) |
| ALT (U/L) | 308.81±553.87 | 188.24±305.56 | 285.25±480.10 |
| AST (U/L) | 211.45±301.69 | 207.67±289.17 | 213.49±296.89 |
| Severe ADR, n (%) | 24(24.00) | 20(33.30) | 44(27.50) |
| Recovery | 23(23.00) | 18(30.00) | 41(25.63) |
| Improved | 66(66.00) | 32(53.33) | 98(61.24) |
| Unchanged | 1(1.00) | 3(5.00) | 4(2.50) |
| Sequelae | 0(0.00) | 1(1.67) | 1(0.63) |
| Unknown | 10(10.00) | 6(10.00) | 16(10.00) |

**Table S2 Identification and trends of change of susceptibility-related metabolites**

| **No.** | **TR (min)** | **m/z** | **Formula** | **Metabolites** | **FC** | **VIP** | **Pathway** |
| --- | --- | --- | --- | --- | --- | --- | --- |
| **ESI^-^** |  |  |  |  |  |  |  |
| 1 | 1.22 | 94.0418 | C6H6O | Phenol | 1.9560 | 1.84957 | Tyrosine metabolism |
| 2 | 1.15 | 125.0141 | C2H7NO3S | Taurine | 1.7208 | 2.16981 | Primary bile acid biosynthesis |
| 3 | 1.09 | 155.0689 | C6H9N3O2 | L-Histidine | 2.6634 | 1.30676 | Histidine metabolism |
| 4 | 1.5 | 165.079 | C9H11NO2 | L-Phenylalanine | 1.541 | 1.21014 | Phenylalanine metabolism |
| 5 | 1.2 | 176.0307 | C6H8O6 | Ascorbate | 1.5375 | 1.03037 | Glutathione metabolism |
| 6 | 1.27 | 192.0276 | C6H8O7 | Citric acid | 2.0427 | 1.95883 | TCA cycle |
| 7 | 1.23 | 193.0731 | C10H11NO3 | Phenylacetylglycine | 1.7977 | 1.29922 | Phenylalanine metabolism |
| 8 | 0.96 | 196.0427 | C9H8O5 | 3-(3,4-Dihydroxyphenyl)-  pyruvate | 2.1458 | 1.14355 | Tyrosine metabolism |
| 9 | 1.08 | 200.0295 | C8H8O6 | 4-Fumarylacetoacetic acid | 1.5034 | 1.41559 | Tyrosine metabolism |
| 10 | 2.18 | 204.0897 | C11H12N2O2 | L-Tryptophan | 1.6203 | 1.32726 | Tryptophan metabolism |
| 11 | 16.91 | 364.2165 | C21H32O5 | Tetrahydrocortisone | 2.2577 | 2.14288 | Steroid hormone biosynthesis |
| 12 | 16.91 | 366.2183 | C21H34O5 | Cortolone | 2.3481 | 1.38482 | Steroid hormone biosynthesis |
| 13 | 17.5 | 366.2339 | C21H34O5 | Tetrahydrocortisol | 3.1695 | 1.46603 | Steroid hormone biosynthesis |
| 14 | 18.46 | 368.2481 | C21H36O5 | Cortol | 2.9092 | 1.30826 | Steroid hormone biosynthesis |
| 15 | 6.28 | 465.3076 | C26H43NO6 | Glycocholic acid | 0.1674 | 1.73628 | Primary bile acid biosynthesis |
| 16 | 12.57 | 466.3105 | C27H46O4S | Cholesterol sulfate | 0.4245 | 1.76066 | Steroid hormone biosynthesis |
| 17 | 21.82 | 588.2902 | C33H40N4O6 | D-Urobilin | 3.2274 | 1.61762 | Porphyrin and chlorophyll metabolism |
| ESI^+^ |  |  |  |  |  |  |  |
| 1 | 2.96 | 117.0567 | C8H7N | Indole | 1.6455 | 1.26596 | Tryptophan metabolism |
| 2 | 1.12 | 131.0697 | C4H9N3O2 | Creatine | 1.5439 | 1.37464 | Arginine and proline metabolism |
| 3 | 1.69 | 165.1192 | C10H15NO | Hordenine | 1.5314 | 2.32932 | Tyrosine metabolism |
| 4 | 2.96 | 187.0625 | C11H9NO2 | Indolepyruvate | 1.6643 | 3.69381 | Tryptophan metabolism |
| 5 | 11.28 | 317.2917 | C18H39NO3 | Phytosphingosine | 1.8967 | 2.35114 | Sphingolipid metabolism |
| 6 | 18.58 | 805.5616 | C42H79NO13 | Lactosylceramide (d18:1/12:0) | 2.1618 | 3.54725 | Sphingolipid metabolism |

**Table S3 Identification and trends of change of liver injury-related metabolites**

| **No.** | **TR (min)** | **m/z** | **Formula** | **Metabolites** | **FC** | **VIP** | **Pathway** |
| --- | --- | --- | --- | --- | --- | --- | --- |
| **ESI^-^** |  |  |  |  |  |  |  |
| 1 | 11.83 | 191.0759 | C7H13NO5 | 2-Amino-3,7-dideoxy-D-  threo-hept-6-ulosonic acid | 3.1440 | 1.45344 | Phenylalanine, tyrosine, and tryptophan biosynthesis |
| 2 | 8.07 | 213.008 | C4H8NO7P | L-Aspartyl-4-phosphate | 10.0760 | 2.61825 | Lysine biosynthesis |
| 3 | 11.73 | 301.2065 | C18H39NO2 | Sphinganine | 1.8300 | 1.14474 | Sphingolipid metabolism |
| 4 | 4.79 | 306.0706 | C15H14O7 | Leucocyanidin | 1.6810 | 1.18603 | Biosynthesis of phenylpropanoids |
| 5 | 10.68 | 354.2512 | C20H34O5 | 11,14,15-THETA | 1.9559 | 1.80174 | Arachidonic acid metabolism |
| 6 | 10.68 | 354.254 | C20H34O5 | 11b-PGF2a | 1.9342 | 1.34553 | Arachidonic acid metabolism |
| 7 | 10.67 | 354.2611 | C20H34O5 | 8-Isoprostane | 1.9518 | 1.71873 | Arachidonic acid metabolism |
| 8 | 9.83 | 354.9743 | C20H34O5 | Prostaglandin F2α | 17.7810 | 1.43804 | Arachidonic acid metabolism |
| 9 | 10 | 354.9744 | C20H34O5 | Trioxilin A3 | 2.5520 | 1.35213 | Arachidonic acid metabolism |
| 10 | 13.49 | 501.2845 | C32H39NO4 | LysoPE(0:0/20:4(5Z,8Z,11Z,14Z)) | 1.7761 | 1.63928 | Bile secretion |
| 11 | 17.19 | 805.5588 | C42H79NO13 | Lactosylceramide (d18:1/12:0) | 1.8267 | 1.26608 | Sphingolipid metabolism |
| 12 | 17.64 | 815.6836 | C47H94NO7P | PE(O-18:1(1Z)/20:4(5Z,8Z,11Z,14Z)) | 0.4235 | 1.92957 | Glycerophospholipid metabolism |
| 13 | 1.08 | 117.0772 | C5H11NO2 | L-Valine | 1.7307 | 3.86402 | Valine, leucine and isoleucine degradation |
| **ESI^-^** |  |  |  |  |  |  |  |
| 1 | 1.28 | 154.0265 | C7H6O4 | Gentisic acid | 2.4875 | 1.43010 | Tyrosine metabolism |
| 2 | 1.27 | 154.0353 | C6H6N2O3 | Imidazol-5-yl-pyruvate | 2.3143 | 1.52210 | Histidine metabolism |
| 3 | 2.04 | 202.03 | C11H6O4 | Bergaptol | 3.4536 | 2.93542 | Biosynthesis of phenylpropanoids |
| 4 | 17.26 | 680.6642 | C47H84O2 | CE (16:1(9Z)) | 1.7645 | 4.07171 | Bile secretion |
